# Cardioselective versus Non-Cardioselective Beta-Blockers and Outcomes in Patients with Atrial Fibrillation and Chronic Obstructive Pulmonary Disease

**DOI:** 10.3390/jcm12093063

**Published:** 2023-04-23

**Authors:** Dimitra Vlachopoulou, Charalampos Balomenakis, Anastasios Kartas, Athanasios Samaras, Andreas S. Papazoglou, Dimitrios V. Moysidis, Konstantinos Barmpagiannos, Melina Kyriakou, Anastasios Papanastasiou, Amalia Baroutidou, Ioannis Vouloagkas, Apostolos Tzikas, George Giannakoulas

**Affiliations:** 1 First Department of Cardiology, AHEPA University Hospital, School of Medicine, Faculty of Health Sciences, Aristotle University of Thessaloniki, 546 36 Thessaloniki, Greece; 2 Interbalkan European Medical Center, Asklipiou 10, 555 35 Thessaloniki, Greece

**Keywords:** cardioselective beta-blockers, non-cardioselective beta-blockers, beta-blockers, atrial fibrillation, chronic obstructive pulmonary disease, mortality

## Abstract

**Background:** Atrial fibrillation (AF) and chronic obstructive pulmonary disease (COPD) have been independently associated with increased mortality; however, there is no evidence regarding beta-blocker cardioselectivity and long-term outcomes in patients with AF and concurrent COPD. **Methods:** This post hoc analysis of the MISOAC-AF randomized trial (NCT02941978) included patients hospitalized with comorbid AF. At discharge, all patients were classified according to the presence of COPD; patients with COPD on beta-blockers were classified according to beta-blocker cardioselectivity. Adjusted hazard ratios (aHRs) were calculated by using multivariable Cox regression models. The primary outcome was all-cause mortality, and the secondary outcomes were cardiovascular mortality and hospitalizations. **Results:** Of 1103 patients with AF, 145 (13%) had comorbid COPD. Comorbid COPD was associated with an increased risk of all-cause (aHR, 1.33; 95% confidence interval (CI), 1.02 to 1.73) and cardiovascular mortality (aHR 1.47; 95% CI, 1.10 to 1.99), but not with increased risk of hospitalizations (aHR 1.10; 95% CI, 0.82 to 1.48). The use of cardioselective versus non-cardioselective beta-blockers was associated with similar all-cause mortality (aHR 1.10; 95% CI, 0.63 to 1.94), cardiovascular mortality (aHR 1.33; 95% CI, 0.71 to 2.51), and hospitalizations (aHR 1.65; 95% CI 0.80 to 3.38). **Conclusions:** In recently hospitalized patients with AF, the presence of COPD was independently associated with increased risk of all-cause and cardiovascular mortality. No difference between cardioselective and non-cardioselective beta-blockers, regarding clinical outcomes, was identified.

## 1. Introduction

Atrial fibrillation (AF) is the most commonly treated arrhythmia [1], and is associated with increased morbidity and mortality [2,3]. It is estimated that up to 14 million people will be affected by AF in Europe by 2060 [4]. The prevalence of chronic obstructive pulmonary disease (COPD) in AF patients is as high as 25% among different studies [5,6,7,8,9,10,11,12,13,14]. Both diseases share common risk factors and pathophysiological mechanisms, including pulmonary hypertension, hypoxia, oxidative stress, and inflammation [15,16,17].

Beta-blockers (BB) are indicated for rate control in patients with AF [18]. Contrary to previous years [19], the use of BBs in COPD patients is not contraindicated, according to the 2022 report of the Global Initiative for the Diagnosis Management and Prevention of Chronic Obstructive Pulmonary Disease [20]. Non-cardioselective BBs block both beta-1- and beta-2-adrenergic receptors, which can lead to a wider range of pharmacodynamic effects compared to cardioselective BBs. Among their effects, one that plays an important role in the prescribing dilemma of patients with COPD is the potential bronchoconstriction as a consequence of the beta-2-adrenergic receptor blockade in the lungs. This effect is considered to be rather problematic in individuals with preexisting lung disease. Conversely, cardioselective BBs have an at least 20 times greater affinity for beta-1-receptors than for beta-2-receptors and theoretically should be associated with a considerably lower risk of bronchoconstriction [21].

Both cardioselective and non-cardioselective BBs are absorbed from the gastrointestinal tract and reach peak plasma concentrations within 1–3 h, reducing, thereby, the effects of adrenaline and noradrenaline, and lowering heart rate, blood pressure, and heart contractility. They are primarily metabolized in the liver (i.e., by the cytochrome P450 system), and are mainly eliminated through renal excretion. The metabolic pathways and elimination (i.e., the “pharmacokinetics”) of BBs can vary depending on the specific drug, which might affect their duration of action and the potential for drug–drug interactions [22].

Nonetheless, selective beta-blockades with beta-1-blockers have been associated with a survival advantage in patients with COPD and concurrent chronic heart failure (HF) or myocardial infarction [23,24]. However, there is no real-world evidence concerning BB cardioselectivity and long-term outcomes in patients with concurrent AF and COPD.

With regard to the above, we conducted a retrospective analysis on a cohort of patients discharged from the hospital with AF. We examined the association of comorbid COPD with mortality and hospitalizations. In the subset of patients with AF and COPD, we explored the association of BB cardioselectivity with these outcomes.

## 2. Materials and Methods

### 2.1. Study Design

This observational study constitutes a post hoc analysis of the randomized controlled MISOAC-AF trial (Motivational Interviewing to Support Oral Anti Coagulation Adherence in patients with Atrial Fibrillation; ClinicalTrials.gov identifier: NCT02941978). Briefly, the MISOAC-AF trial examined the effect of a combined motivational–educational intervention on adherence to prescribed oral anticoagulation therapy in patients with AF following an acute hospitalization. The design, the selection criteria, and the main results of the MISOAC-AF trial have been previously published [25,26]. The MISOAC-AF trial conforms to the ethical principles of the Declaration of Helsinki and was approved by the institutional review board and ethics committee of Aristotle University of Thessaloniki [27]. All participants provided written informed consent for participation in the study.

### 2.2. Data and Population Sources

Our cohort consisted of patients over the age of 18, who were hospitalized in a single-center academic hospital (AHEPA University General Hospital of Thessaloniki) with a primary or secondary diagnosis of AF. After hospital discharge, patients were followed up from December 2015 to June 2018. The database of the MISOAC-AF trial included information on COPD status and prescription of BBs at discharge. Prescription of BBs was further classified according to BB selectivity (cardioselective and non-cardioselective BBs). Baseline demographic; clinical; laboratory; and echocardiographic data, as well as comorbidities; medication; and prospective clinical outcomes were extracted from the MISOAC-AF database for the purpose of this study.

### 2.3. Definition of Diagnoses and Covariates

The diagnosis of AF was established through electrocardiograms showing irregular RR intervals and no distinct P waves for more than 30 s. Diagnosis of COPD was determined through identification of prescribed inhalation therapy (short-acting (SABA) and long-acting (LABA) beta2-agonists and inhaled corticosteroids). Data concerning medication therapy of COPD were acquired, either from discharge letters or through the Greek electronic medical prescription system.

### 2.4. Study Outcomes and Follow-Up

The primary outcome was all-cause mortality. Secondary outcomes were cardiovascular mortality and hospitalizations after discharge. Cardiovascular mortality was predefined in the MISOAC-AF trial [26].

Follow-up was conducted by independent researchers annually, through phone calls or in-person interviews. Mortality during the MISOAC-AF trial was ascertained through the Greek Civil Registration System. The follow-up period was extended until May 2020.

### 2.5. Statistical Analysis

The student’s *t*-test and Wilcoxon–Mann–Whitney test were used for continuous numerical variables, according to the normality of their distribution. Chi-squared tests or Fisher’s exact tests were used for categorical variables. For the survival analysis, Kaplan–Meier curves were constructed and log-rank tests were performed. Cox regression models were calculated in both comparisons to acquire an adjusted hazard ratio (aHR) for the effects of the explanatory variables. The maximum number of independent variables, that could be included in the final model, was determined by the rule of one predictor variable for ten outcomes of interest. Variable selection was based on literature research, and a *p*-value cut-off of 0.1 was set in univariate analysis. Variables used for adjustment were age, BBs, diabetes mellitus, left ventricular ejection fraction (LVEF) ≤ 40%, and history of coronary artery disease (CAD) as well as prior stroke. To ensure no violation of the assumptions of the Cox regression models, Schoenfeld residuals were examined. The level of significance (α) was set to 5%. Two-tailed *p*-values were calculated. The multiple imputations method was performed to impute any missing data. The number of imputations was 20 for 10–30% missing data and 40 for >50%. In regression analyses, the results were pooled into one point estimate according to Rubin’s rule. Additionally, we also performed survival Cox regression analyses based on the initial dataset (with missing non-imputed variables). The results derived from these analyses are shown in the Supplementary Material. For the statistical analysis, the R programming language (version 4.0.5) and the RStudio integrated development environment (version 1.4.1106) were utilized.

## 3. Results

### 3.1. Patient Characteristics

The study population consisted of 1103 patients with AF, of whom 145 (13.1%) had a concomitant diagnosis of COPD. In general, patients with COPD were more frequently smokers, had a higher CHA_2_DS_2_-VASc score or a prior history of stroke and similar prescription rates of BBs and oral anticoagulants (OAC) compared to non-COPD patients (Supplementary Materials, Table S1). Of patients with AF and COPD, 85.5% were treated with BBs and 59.7% with cardioselective BBs (Table 1). Interestingly, the male gender, a history of CAD, prior myocardial infarction, diabetes mellitus, and prior stroke were more prevalent in the non-cardioselective group. No significant difference was noted in the prescription rates of inhaled beta-agonists or corticosteroids.

### 3.2. AF Patients with COPD

**Figure 1:**
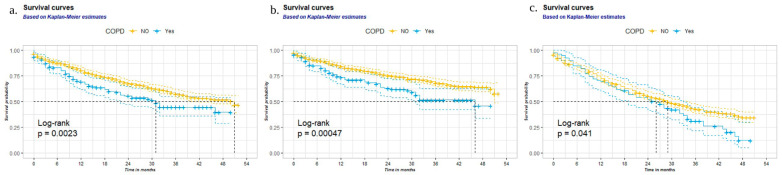
Survival curves of AF patients with and without COPD (univariate analysis): (**a**) all-cause mortality rates; (**b**) cardiovascular mortality rates; and (**c**) hospitalization rates during follow-up.

Over a median 2.7-year follow-up period, 412 of 1102 patients (37.4%) died. A univariable analysis showed a significantly higher all-cause mortality rate in COPD patients than in non-COPD patients (unadjusted HR: 1.50; 95% CI: 1.15 to 1.94, *p* = 0.002 by log-rank test) (Figure 1a). Significance was retained after adjustment for covariates (aHR: 1.33; 95% CI: 1.02 to 1.73) (Table 2).

A cardiovascular cause of mortality was attributed to 315 of 427 (73.3%) deaths documented during the follow-up period. Of patients with AF and COPD on BB therapy, the rate of death was 47.9% (57 deaths in 119 patients). COPD was a significant prognostic factor for cardiovascular mortality in AF (unadjusted HR: 1.68; 95% CI: 1.25 to 2.25 and aHR: 1.47; 95% CI: 1.10 to 1.99) (Table 2). That is also validated graphically (*p* < 0.001 by log-rank test) (Figure 1b). During the follow-up period, no significant difference was found in hospital admission rates between the COPD and non-COPD groups (unadjusted HR: 1.34; 95% CI: 1.01 to 1.77, and aHR: 1.10; 95% CI: 0.82 to 1.48) (Table 2, Figure 1c).

When we conducted the aforementioned Cox regression analyses, based on the initial dataset (with significant number of missing non-imputed variables), COPD was not an independent predictor of those clinical outcomes, despite its univariate association with increased risk of mortality, CV mortality or hospitalizations during follow-up (Supplementary Table S2).

### 3.3. Beta-Blocker Cardioselectivity

**Figure 2:**
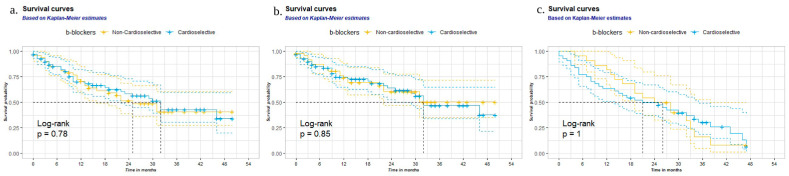
Survival curves of AF patients with cardioselective and non-cardioselective b-blockers (univariate analysis): (**a**) all-cause mortality rates, (**b**) cardiovascular mortality rates, and (**c**) hospitalization rates during follow-up.

During the follow-up period, a total of 57 deaths (52.8%) occurred in COPD patients on BB therapy. At the time of discharge, 71 patients were treated with cardioselective BBs and 32 of them died (45.1%), as compared with 25 of 48 patients treated with non-cardioselective BBs (52.1%). Statistical analyses did not reach significance on all-cause mortality between cardioselective and non-cardioselective BBs (unadjusted HR: 0.93; 95% CI: 0.55 to 1.57, and aHR: 1.10; 95% CI: 0.63 to 1.94 (Table 3), *p* = 0.78 via the log-rank test (Figure 2a)).

The risk of cardiovascular mortality was similar between cardioselective and non-cardioselective BBs (unadjusted HR: 1.06; 95% CI: 0.59 to 1.90 and aHR: 1.33; 95% CI: 0.71 to 2.51 (Table 3), *p* = 0.85 via the log-rank test (Figure 2b)). No significant difference was found in hospitalization rates either (unadjusted HR: 0.99; 95% CI: 0.55 to 1.79, and aHR: 1.65; 95% CI: 0.80 to 3.38) (Table 3, Figure 2c).

The cardioselectivity of BBs was also not significantly associated with the occurrence of these clinical outcomes according to the analyses performed, based on the initial dataset (with a significant number of missing non-imputed variables) (Supplementary Table S2).

## 4. Discussion

This retrospective study examined the prognostic implications of COPD and BB cardioselectivity on the clinical course of AF in hospitalized patients with comorbid COPD followed up after their discharge. We validated the high prognostic significance of COPD comorbidity in all-cause and cardiovascular mortality of AF patients. No significant association regarding BB cardioselectivity and clinical outcomes in AF patients with comorbid COPD was demonstrated. To our knowledge, this is the first study investigating the association of BB cardioselectivity, in patients with AF and COPD, with clinical outcomes.

The prevalence of concomitant COPD in our population (13.1%) was within the rate published in the current literature. The later ranges were between 10.8% and 25% [5,8,12,13,14]. Our findings revealed a similar prescription of BBs in the COPD and non-COPD groups. The administration of BBs has been shown to be less common in cases of AF–COPD comorbidity than in AF alone in most studies [5,13,14]. Hence, our study may also imply that reservations against BB use in AF–COPD comorbidity are decreasing.

Our findings are in accordance with published data concerning mortality in AF-COPD comorbidity. According to a recently published meta-analysis, the odds of all-cause mortality were approximately 2 to 3 times higher in AF patients with COPD compared to patients with AF alone, while the odds of cardiovascular mortality were up to 143% higher in cases of COPD comorbidity [14]. Our findings did not reach a statistically significant difference concerning hospitalization rates in AF–COPD comorbidity, in comparison with AF alone. In the case of COPD comorbidity, Durheim et al. (2018) found higher rates of cardiovascular hospitalizations, while Méndez-Bailón et al. (2017) reported higher AF-related hospitalization rates [6,9].

Moreover, our study found no association between BBs and long-term patient outcomes. Our findings are in accordance with the prospective cohort study of Proietti et al. (2016) and a recently published meta-analysis, which did not identify BB use as a risk factor for clinical outcomes in patients with concurrent AF and COPD [13,14]. On the contrary, Durheim et al. (2018) found a decreased incidence of myocardial infarction in patients on BBs [6]. In addition, Rodriguez et al. (2019) suggested BB intake as a prognostic factor for lower mortality [12].

The comparison between cardioselective and non-cardioselective BBs did not reach statistical significance with regard to long-term outcomes. The use of BBs and their cardioselectivity, in patients with COPD and cardiovascular diseases, has been previously investigated. A meta-analysis of Yang et al. (2020), on concurrent COPD and cardiovascular diseases, showed no association of cardioselective and non-cardioselective BBs with the acute exacerbation of COPD events and heart rate control [28]. However, most studies have focused on other cardiovascular diseases, such as HF and myocardial infarction, and no prior studies have evaluated the association between BB cardioselectivity and all-cause mortality in AF-COPD patients [28]. According to the 2021 European Society of Cardiology Guidelines, non-dihydropyridine calcium channel blockers (NDCCBs) are the first-line therapy for cases of AF with concurrent severe COPD or asthma [18]. However, a recent study has demonstrated an association of BBs, cardioselective and non-cardioselective, with lower mortality rates than NDCCBs in AF patients with obstructive lung disease [29]. Based on these findings, we expect that more studies will focus on the investigation of BBs and their cardioselectivity for rate control in patients with AF and concomitant COPD.

The strengths of this study include the long follow-up period and the availability of information concerning medication and inhalation therapy. COPD medications, such as inhalation therapy and corticosteroids, are not often mentioned in studies examining AF–COPD comorbidity.

However, the sample size was relatively small regarding the comparison between cardioselective and non-cardioselective BBs and there was a high percentage of missing values regarding the outcome of hospitalization (40–50%). Moreover, COPD diagnosis was not based on GOLD criteria, and identifying patients with COPD using an already-known spirometry-based diagnosis might have been more accurate than just using a treatment-based diagnostic criterion. Furthermore, the severity of COPD was not taken into account in our analysis. Additionally, changes in medication during follow-up were not captured. The documentation of any drug-related adverse events or a sub-analysis on the precise cause of mortality, or the individual BB drug and dose, could not be achieved in our study due to the unavailability of the required data. Furthermore, the study population consisted of patients who likely live in an urban environment or have access to a university hospital, and have been hospitalized due to a cardiological cause.

## 5. Conclusions

In recently hospitalized patients with AF, the presence of COPD comorbidity was associated with increased all-cause and cardiovascular mortality. No significant difference of cardioselective and non-cardioselective BB use with the clinical outcomes of AF–COPD patients was identified. Further prospective trials are warranted prior to reaching definite conclusions for the optimal medical management of this challenging comorbidity.

## Figures and Tables

**Table 1:** Baseline characteristics of patients with AF and COPD taking BBs.

Patient Baseline Characteristics				
Characteristic	N	Non-Cardioselective BB, N = 48	Cardioselective BB, N = 71	*p*-Value ^a^
Age, median (IQR ^b^)	117	77 (12)	76 (11)	>0.9
Gender, n/N (%)	119			**<0.01**
Male		37/48 (77%)	36/71 (51%)	
Female		11/48 (23%)	35/71 (49%)	
BMI ^b^, median (IQR ^b^)	116	28.1 (6.5)	27.6 (6.7)	0.8
Smoking, n/N (%)	116	36/47 (77%)	47/69 (68%)	0.3
LVEF, n/N (%)	117			0.5
>40%		31/48 (65%)	49/69 (71%)	
≤40%		17/48 (35%)	20/69 (29%)	
CAD, n/N (%)	117	33/47 (70%)	25/70 (36%)	**<0.001**
Prior MI ^b^, n/N (%)	117	22/47 (47%)	14/70 (20%)	**<0.01**
AH ^b^, n/N (%)	117	42/48 (88%)	60/69 (87%)	>0.9
Diabetes Mellitus, n/N (%)	119	26/48 (54%)	22/71 (31%)	**0.011**
Dylipidemia, n/N (%)	118	26/47 (55%)	37/71 (52%)	0.7
CKD ^b^, n/N (%)	117	13/47 (28%)	13/70 (19%)	0.2
Prior Stroke, n/N (%)	117	15/48 (31%)	10/69 (14%)	**0.03**
AF type, n/N (%)	115			0.7
Non-valvular		5/48 (10.4%)	4/71 (5.6%)	
Valvular		43/48 (89.6%)	67/71 (94.4%)	
OACs ^b^, n/N (%)	115			0.2
no		6/47 (13%)	6/68 (8.8%)	
VKA ^b^		19/47 (40%)	19/68 (28%)	
NOAC ^b^		22/47 (47%)	43/68 (63%)	
Anti-PLTs ^b^, n/N (%)	111			0.2
No		43/47 (91%)	53/64 (83%)	
Aspirin		3/47 (6.4%)	2/64 (3.1%)	
Clopidogrel		0/47 (0%)	3/64 (4.7%)	
Aspirin and clopidogrel		1/47 (2.1%)	6/64 (9.4%)	
CHA_2_DS_2__VAS_C_,n/N (%)	118			0.2
Low risk		1/47 (2.1%)	6/71 (8.5%)	
High risk		46/47 (98%)	65/71 (92%)	
Corticosteroids, n/N (%)	114			0.6
No		27/46 (59%)	38/68 (56%)	
inhalative		17/46 (37%)	29/68 (43%)	
per os		2/46 (4.3%)	1/68 (1.5%)	
Inhalatives, n/N (%)	114			0.5
No		22/46 (48%)	34/68 (50%)	
SABA		2/46 (4.3%)	3/68 (4.4%)	
LABA		20/46 (43%)	31/68 (46%)	
SABA and LABA		2/46 (4.3%)	0/68 (0%)	

^a^ Statistical tests performed: Wilcoxon rank-sum test; chi-square test of independence; Fisher’s exact test. ^b^ BMI = body mass index, IQR = interquartile range, MI = myocardial infarction, AH = arterial hypertension, CKD = chronic kidney disease, OAC = oral anticoagulant, VKA = vitamin K antagonist, NOAC = new oral anticoagulant, and PLTs = platelets. *p*-values marked in bold indicate statistically significant tests (*p* < 0.05).

**Table 2:** Univariate and multivariate analyses for patients with AF with and without COPD.

	All-Cause Mortality				Cardiovascular Mortality				Hospitalizations			
	Univariate Analysis		Multivariate Analysis		Univariate Analysis		Multivariate Analysis		Univariate Analysis		Multivariate Analysis	
	HR (95%CI)	*p*-Value	aHR (95% CI)	*p*-Value	HR (95% CI)	*p*-Value	aHR (95% CI)	*p*-Value	HR (95% CI)	*p*-Value	aHR (95% CI)	*p*-Value
Age	1.04 (1.03, 1.05)	<0.001	1.04 (1.03, 1.05)	**<0.001**	1.04 (1.03, 1.05)	<0.001	1.04 (1.03, 1.06)	**<0.001**	1.04 (1.03, 1.05)	<0.001	1.04 (1.03, 1.06)	**<0.001**
Diabetes mellitus	1.59 (1.31, 1.94)	<0.001	1.44 (1.18, 1.77)	**<0.001**	1.70 (1.35, 2.13)	<0.001	1.49 (1.17, 1.88)	**<0.01**	1.26 (1.02, 1.55)	0.03	1.15 (0.92, 1.43)	0.22
LVEF ≤ 40%	2.80 (2.28, 3.44)	<0.001	2.85 (2.30, 3.55)	**<0.001**	3.45 (2.74, 4.35)	<0.001	3.41 (2.67, 4.35)	**<0.001**	1.76 (1.38, 2.24)	<0.001	1.13 (0.86, 1.50)	0.38
CAD	155 (0.91, 2.62)	<0.001	1.04 (0.84, 1.29)	0.7	1.56 (1.24, 1.96)	<0.001	1.08 (0.85, 1.38)	0.52	1.25 (0.96, 1.62)	0.1	0.95 (0.76, 1.20)	0.67
BB	0.93 (0.55, 1.57)	0.06	1.08 (0.85, 1.37)	0.54	1.41 (1.06, 1.88)	0.02	1.16 (0.87, 1.56)	0.31	n.s.		n.a.	
COPD	1.50 (1.15, 1.94)	<0.01	1.33 (1.02, 1.73)	**0.03**	1.68 (1.25, 2.25)	<0.001	1.47 (1.10, 1.99)	**0.02**	1.34 (1.01, 1.77)	<0.04	1.10 (0.82, 1.48)	0.52
Prior stroke	n.s.		n.a.		n.s.		n.a.		1.14 (0.93, 1.40)	0.2	0.82 (0.60, 1.12)	0.21

(a) HR: (adjusted) hazard ratio, CI: confidence intervals, n.s.: non-significant, and n.a.: not applicable. *p*-values marked in bold indicate statistically significant tests (*p* < 0.05).

**Table 3:** Univariate and multivariate analysis for patients with AF and COPD on BB therapy.

	All-Cause Mortality				Cardiovascular Mortality				Hospitalizations			
	Univariate Analysis		Multivariate Analysis		Univariate Analysis		Multivariate Analysis		Univariate Analysis		Multivariate Analysis	
	HR (95% CI)	*p*-Value	aHR (95% CI)	*p*-Value	HR (95% CI)	*p*-Value	aHR (95% CI)	*p*-Value	HR (95% CI)	*p*-Value	aHR (95% CI)	*p*-Value
Diabetes mellitus	1.16 (0.70, 1.94)	0.57	109 (0.63, 1.88)	0.76	1.20 (0.67, 2.17)	0.53	1.17 (0.64, 2.15)	0.6	1.16 (0.66, 2.03)	0.6	1.41 (0.77, 2.58)	0.26
LVEF ≤ 40%	2.88 (1.68, 4.94)	<0.01	2.85 (1.51, 5.36)	**<0.01**	3.34 (1.84, 6.07)	<0.01	3.20 (1.59, 6.44)	**<0.01**	1.69 (0.90, 3.16)	0.1	1.03 (0.47, 2.28)	0.94
Age	1.04 (1.01, 1.07)	0.02	1.05 (1.01, 1.08)	**0.02**	1.04 (1.00, 1.07)	0.05	1.04 (1.00, 1.08)	**0.05**	1.04 (1.00, 1.07)	0.05	1.04 (1.00, 1.08)	**0.05**
CAD	155 (0.91, 2.62)	0.1	1.04 (0.55, 1.97)	0.9	1.75 (0.97, 3.16)	0.06	1.20 (0.58, 2.43)	0.61	1.21 (0.70, 2.10)	0.49	1.27 (0.62, 2.62)	0.51
Cardioselective BB	0.93 (0.55, 1.57)	0.78	1.10 (0.63, 1.94)	0.73	1.06 (0.59, 1.9)	0.85	1.33 (0.71, 2.51)	0.37	0.99 (0.55, 1.79)	0.98	1.65 (0.80, 3.38)	0.17
Prior stroke	n.s.		n/a		n.s.		n/a		0.71 (0.35, 1.47)	0.36	0.79 (0.36, 1.77)	0.56

(a) HR: (adjusted) hazard ratio, CI: confidence intervals, n.s.: non-significant, and n.a.: not applicable. *p*-values marked in bold indicate statistically significant tests (*p* < 0.05).

## Data Availability

Data are available from George Giannakoulas (email: ggiannakoulas@auth.gr) upon reasonable request and with permission of AHEPA University Hospital.

## Supplementary Materials

The following supporting information can be downloaded at: https://www.mdpi.com/article/10.3390/jcm12093063/s1, Table S1: Baseline characteristics grouped by COPD status, Table S2: Outcomes of the performed Cox regression analyses in the initial dataset without imputation of the missing data.

## References

[1] Center for Disease Control and Prevention (CDC) Atrial Fibrillation 2020. https://www.cdc.gov/heartdisease/atrial_fibrillation.htm.

[2] Ruddox V., Sandven I., Munkhaugen J., Skattebu J., Edvardsen T., Otterstad J.E. (2017). Atrial fibrillation and the risk for myocardial infarction, all-cause mortality and heart failure: A systematic review and meta-analysis. Eur. J. Prev. Cardiol..

[3] Odutayo A., Wong C.X., Hsiao A.J., Hopewell S., Altman D.G., Emdin C.A. (2016). Atrial fibrillation and risks of cardiovascular disease, renal disease, and death: Systematic review and meta-analysis. BMJ.

[4] di Carlo A., Bellino L., Consoli D., Mori F., Zaninelli A., Baldereschi M., Cattarinussi A., D’Alfonso M.G., Gradia C., Sgherzi B. (2019). Prevalence of atrial fibrillation in the Italian elderly population and projections from 2020 to 2060 for Italy and the European Union: The FAI Project. Europace.

[5] Durheim M.T., Cyr D.D., Lopes R.D., Thomas L.E., Tsuang W.M., Gersh B.J., Held C., Wallentin L., Granger C.B., Palmer S.M. (2016). Chronic obstructive pulmonary disease in patients with atrial fibrillation: Insights from the ARISTOTLE trial. Int. J. Cardiol..

[6] Durheim M.T., Holmes D.N., Blanco R.G., Allen L.A., Chan P.S., Freeman J.V., Fonarow G.C., Go A.S., Hylek E.M., Mahaffey K.W. (2018). Characteristics and outcomes of adults with chronic obstructive pulmonary disease and atrial fibrillation. Heart.

[7] Simons S.O., Elliott A., Sastry M., Hendriks J.M., Arzt M., Rienstra M., Kalman J.M., Heidbuchel H., Nattel S., Wesseling G. (2021). Chronic obstructive pulmonary disease and atrial fibrillation: An interdisciplinary perspective. Eur. Heart J..

[8] Angeli F., Reboldi G., Trapasso M., Aita A., Ambrosio G., Verdecchia P. (2019). Detrimental impact of chronic obstructive pulmonary disease in atrial fibrillation: New insights from Umbria atrial fibrillation registry. Medicina.

[9] Méndez-Bailón M., Lopez-de-Andrés A., de Miguel-Diez J., de Miguel-Yanes J.M., Hernández-Barrera V., Muñoz-Rivas N., Lorenzo-Villalba N., Jiménez-García R. (2017). Chronic obstructive pulmonary disease predicts higher incidence and in hospital mortality for atrial fibrillation. An observational study using hospital discharge data in Spain (2004–2013). Int. J. Cardiol..

[10] Camm A.J., Kirchhof P., Lip G.Y., Schotten U., Savelieva I., Ernst S., Van Gelder I.C., Al-Attar N., Hindricks G., Prendergast B. (2010). Guidelines for the management of atrial fibrillation: The Task Force for the Management of Atrial Fibrillation of the European Society of Cardiology (ESC). Europace.

[11] Huang B., Yang Y., Zhu J., Liang Y., Zhang H., Tian L., Shao X., Wang J. (2014). Clinical characteristics and prognostic significance of chronic obstructive pulmonary disease in patients with atrial fibrillation: Results from a multicenter atrial fibrillation registry study. J. Am. Med. Dir. Assoc..

[12] Rodríguez-Mañero M., López-Pardo E., Cordero A., Ruano-Ravina A., Novo-Platas J., Pereira-Vázquez M., Martínez-Gómez Á., García-Seara J., Martínez-Sande J.L., Peña-Gil C. (2019). A prospective study of the clinical outcomes and prognosis associated with comorbid COPD in the atrial fibrillation population. Int. J. Chron. Obstruct. Pulmon. Dis..

[13] Proietti M., Laroche C., Drozd M., Vijgen J., Cozma D.C., Drozdz J., Maggioni A.P., Boriani G., Lip G.Y., EORP-AF Investigators (2016). Impact of chronic obstructive pulmonary disease on prognosis in atrial fibrillation: A report from the EURObservational Research Programme Pilot Survey on Atrial Fibrillation (EORP-AF) General Registry. Am. Heart J..

[14] Romiti G.F., Corica B., Pipitone E., Vitolo M., Raparelli V., Basili S., Boriani G., Harari S., Lip G.Y.H., Proietti M. (2021). Prevalence, management and impact of chronic obstructive pulmonary disease in atrial fibrillation: A systematic review and meta-analysis of 4,200,000 patients. Eur. Heart J..

[15] Goudis C.A. (2017). Chronic obstructive pulmonary disease and atrial fibrillation: An unknown relationship. J. Cardiol..

[16] Vahdatpour C.A., Luebbert J.J., Palevsky H.I. (2020). Atrial arrhythmias in chronic lung disease-associated pulmonary hypertension. Pulm. Circ..

[17] Babapoor-Farrokhran S., Gill D., Alzubi J., Mainigi S.K. (2021). Atrial fibrillation: The role of hypoxia-inducible factor-1-regulated cytokines. Mol. Cell. Biochem..

[18] Hindricks G., Potpara T., Dagres N., Arbelo E., Bax J.J., Blomström-Lundqvist C., Boriani G., Castella M., Dan G.A., Dilaveris P.E. (2021). 2020 ESC Guidelines for the diagnosis and management of atrial fibrillation developed in collaboration with the European Association for Cardio-Thoracic Surgery (EACTS): The Task Force for the diagnosis and management of atrial fibrillation of the European Society of Cardiology (ESC) Developed with the special contribution of the European Heart Rhythm Association (EHRA) of the ESC. Eur. Heart J..

[19] January C.T., Wann L.S., Alpert J.S., Calkins H., Cleveland J.C., Cigarroa J.E., Conti J.B., Ellinor P.T., Ezekowitz M.D., Field M.E. (2014). 2014 AHA/ACC/HRS guideline for the management of patients with atrial fibrillation: A report of the American College of Cardiology/American Heart Association Task Force on practice guidelines and the Heart Rhythm Society. Circulation.

[20] Global Initiative for Chronic Obstructive Pulmonary Disease (2022). Global Strategy for the Diagnosis, Management, and Prevention of Chronic Obstructive Pulmonary Disease (2022 Report). https://goldcopd.org/2022-gold-reports.

[21] Bennett M., Chang C.L., Tatley M., Savage R., Hancox R.J. (2021). The safety of cardioselective β_1_-blockers in asthma: Literature review and search of global pharmacovigilance safety reports. ERJ Open Res..

[22] Ågesen F.N., Weeke P.E., Tfelt-Hansen P., Tfelt-Hansen J. (2019). Pharmacokinetic variability of beta-adrenergic blocking agents used in cardiology. Pharmacol. Res. Perspect..

[23] Chung C.M., Lin M.S., Chang S.T., Wang P.C., Yang T.Y., Lin Y.S. (2022). Cardioselective versus nonselective β-Blockers after myocardial infarction in adults with chronic obstructive pulmonary disease. Mayo Clin. Proc..

[24] Dransfield M.T., Voelker H., Bhatt S.P., Brenner K., Casaburi R., Come C.E., Cooper J.A.D., Criner G.J., Curtis J.L., Han M.K. (2019). Metoprolol for the prevention of acute exacerbations of COPD. N. Engl. J. Med..

[25] Samaras A., Kartas A., Vasdeki D., Dividis G., Forozidou E., Fotos G., Kotsi E., Paschou E., Tsoukra P., Goulas I. (2020). Rationale and design of a randomized study comparing Motivational Interviewing to Support Oral Anticoagulation adherence versus usual care in patients with nonvalvular atrial fibrillation: The MISOAC-AF trial. Hellenic J. Cardiol..

[26] Tzikas A., Samaras A., Kartas A., Vasdeki D., Fotos G., Dividis G., Paschou E., Forozidou E., Tsoukra P., Kotsi E. (2021). Motivational Interviewing to Support Oral AntiCoagulation adherence in patients with non-valvular Atrial Fibrillation (MISOAC-AF): A randomized clinical trial. Eur. Heart J. Cardiovasc. Pharmacother..

[27] World Medical Association (2013). World Medical Association Declaration of Helsinki: Ethical principles for medical research involving human subjects. JAMA.

[28] Yang Y.L., Xiang Z.J., Yang J.H., Wang W.J., Xu Z.C., Xiang R.L. (2020). Association of β-blocker use with survival and pulmonary function in patients with chronic obstructive pulmonary disease: A systematic review and meta-analysis. Eur. Heart J..

[29] You S.C., An M.H., Yoon D., Ban G.Y., Yang P.S., Yu H.T., Park R.W., Joung B. (2018). Rate control and clinical outcomes in patients with atrial fibrillation and obstructive lung disease. Heart Rhythm.

