# Supplementary material for: Cardioselective versus Non-Cardioselective Beta-Blockers and Outcomes in Patients with Atrial Fibrillation and Chronic Obstructive Pulmonary Disease
Source: J Clin Med. 2023 Apr 23;12(9):3063. doi: 10.3390/jcm12093063 (PMC10179681; doi:10.3390/jcm12093063)
Supplement: Supplementary file 1 [file jcm-12-03063-s001.zip › jcm-2346803-supplementary.pdf]

Supplementary Materials

Supplementary Table S1. Baseline characteristics grouped by COPD status.

Patients' Baseline Characteristics				
**Characteristic**	**N**	**Non-COPD, N = 958**	**COPD, N = 145**	**p-value[a]**
**Age, median (IQR[b])**	1097	76 (14)	77 (12)	0.091
**Gender, n/N (%)**	1102			0.2
Male		513/957 (54%)	86/145 (59%)	
Female		444/957 (46%)	59/145 (41%)	
**BMI[b], median (IQR[b])**	1078	27.8 (6.1)	28.1 (6.7)	0.5
**Smoking, n/N (%)**	1095	560/953 (59%)	101/142 (71%)	**0.005**
**LVEF, n/N (%)**	1022	449/957 (47%)	108/142 (76%)	**0.047**
≤40 %		203/879 (23%)	44/143 (31%)	
>40 %		676/879 (77%)	99/143 (69%)	
**CAD, n/N (%)**	1046	368/904 (41%)	65/141 (46%)	0.3
**Prior MI[b], n/N (%)**	1099	193/956 (20%)	37/143 (26%)	0.12
**AH[b], n/N (%)**	1101	769/958 (80%)	125/143 (87%)	**0.041**
**Diabetes mellitus, n/N (%)**	1103	305/958 (32%)	62/145 (43%)	**0.009**
**Dyslipidemia, n/N (%)**	1102	452/958 (47%)	78/144 (54%)	0.12
**CKD[b], n/N (%)**	1098	128/955 (13%)	36/143 (25%)	**<0.001**
**Prior Stroke, n/N (%)**	1096	138/953 (14%)	32/143 (22%)	**0.015**
**AF type, n/N (%)**	1103			>0.9
Non-Valvular		70/958 (7.3%)	11/145 (7.6%)	
Valvular		888/958 (93%)	134/145 (92%)	
**BB, n/N (%)**	1093	722/949 (76%)	108/144 (75%)	0.8
**OACs[b], n/N (%)**	962			0.10
no		146/824 (18%)	15/138 (11%)	
VKA[b]		236/824 (29%)	47/138 (34%)	
NOAC[b]		442/824 (54%)	76/138 (55%)	
**Anti-PLTs[b], n/N (%)**	935			**0.027**
No		597/803 (74%)	114/132 (86%)	
Aspirin		69/803 (8.6%)	6/132 (4.5%)	
Clopidogrel		58/803 (7.2%)	4/132 (3.0%)	
Aspirin and Clopidogrel		79/803 (9.8%)	7/132 (6.1%)	
**CHA_2DS_2_VASc, n/N (%)**	1075			**0.027**
Low Risk		134/931 (14%)	11/144 (7.6%)	
High Risk		797/931 (86%)	133/144 (92%)	

a. Statistical tests performed: Wilcoxon rank-sum test; chi-square test of independence; b. BMI = Body Mass Index, IQR = Interquartile range, MI = Myocardial infarction, AH = Arterial Hypertension, CKD = Chronic Kidney Disease, OAC = Oral anticoagulant, VKA = Vitamin-K Antagonist, NOAC = New Oral Anticoagulant, PLT = Platelet. P-values in bold indicate statistically significant tests

Supplementary Table S2. Outcomes of the performed Cox regression analyses in the initial dataset without imputation of the missing data

**Outcome assessed**	**Variable**	**Unadjusted HR (95% Cis)**	**p-value**	**Adjusted HR* (95% Cis)**	**p-value**
Death	COPD	1.50 (1.15-1.94)	**0.002**	1.19 (0.91-1.57)	0.20
Death	*Cardioselective BBs*	0.93 (0.55-1.57)	0.78	1.221 (0.70-2.14)	0.48
CV death	COPD	1.68 (1.25-2.25)	**<0.001**	1.25 (0.92-1.70)	0.15
CV death	*Cardioselective BBs*	1.06 (0.59-1.90)	0.85	1.41 (0.76-2.62)	0.27
Hospitalization during FU	COPD	1.34 (1.01-1.77)	**0.04**	1.21 (0.91-1.62)	0.20
Hospitalization during FU	*Cardioselective BBs*	0.99 (0.55-1.79)	0.98	0.93 (0.51-1.71)	0.82

*Variables used for adjustment in the multivariable Cox regression models:
age, body mass index, history of heart failure, coronary artery disease, diabetes mellitus and prior stroke. P-values in bold indicate statistically significant tests.
